# Heatwave types and frequency alter multigenerational ecological response of wheat aphids

**DOI:** 10.1038/s41598-025-13097-x

**Published:** 2025-08-03

**Authors:** Junyu Cao, Kun Xing, Fei Zhao, Weiwei Li

**Affiliations:** 1 Department of Biological and Food Engineering, Lvliang University, Lvliang, 033001 China; 2 Shanxi Key Laboratory of Integrated Pest Management in Agriculture, College of Plant Protection, Shanxi Agricultural University, Taiyuan, 030031 China; 3 Yicheng County Meteorological Bureau of Shanxi Province, Yicheng, 043500 China

**Keywords:** *Sitobion avenae*, Heat stress, Maternal, Offspring, Fitness traits, Ecology, Agroecology, Climate-change ecology

## Abstract

Heatwaves are predicted to become more frequent under climate change. However, how different heatwave types and frequencies are linked to multigenerational ecological response remains unclear. Thus, using three heatwave types (34 °C/180 min, 36 °C/30 min, and 38 °C/10 min) and three frequencies (1, 3, and 5 days), we tested the effects of heat stress on the life history traits of *Sitobion avenae*. Both heatwave type and frequency altered aphid fitness in a generation-specific manner. In maternal generations, at a 1-day frequency, 34℃/180 min exhibited the greatest effects on reproduction and longevity. However, as frequency increased, differences across various heat stress treatments diminished. Conversely, in offspring, 34℃/180 min had minimal effects on demographic parameters. Offspring performance was poorest at the 3-day frequency, indicating that a longer frequency for the mother does not necessarily mean a greater effect on the offspring. Changes in aphid fitness due to heatwave type and frequency are driven by asymmetric effects of heatwaves on aphid life history. Furthermore, understanding how heat stresses affect the life history of aphids is important for predicting the impacts of climate change at the generational level because climate effects on natural populations cannot be predicted simply through changes in mean temperature.

**Supplementary Information:**

The online version contains supplementary material available at 10.1038/s41598-025-13097-x.

## Introduction

Temperature is a crucial factor influencing the survival^1,2^growth and development^3–5^and reproduction of poikilotherm animals like insects^6,7^. However, temperature is rarely stable meaning that insects frequently experience short and extended periods of hot days^8–10^. Global warming increases this instability because high-temperature events are expected to become longer, more variable, intense, and frequent^11–14^. Insects do not passively endure high temperatures but demonstrate active behaviors for thermal regulation^15,16^. For example, when insects are exposed to extremely high temperatures, they rapidly adjust their behaviors and seek shade^16–18^. Moreover, the time it takes insects to respond to high temperatures significantly differs depending on the conditions^19,20^which will lead to variability in the heatwave types (defined as combinations of temperature and duration). Therefore, the effects of heat events of various types and frequencies that do not cause death on the life history traits of insects warrants further investigation.

The environmental temperature experienced by the maternal generation can influence the growth and development of the offspring. For example, when *Bicyclus anynana* lays eggs at higher temperatures, the eggs are smaller, but the survival rate increases, thereby ensuring reproductive success^21^. In *Aphidius ervi*, maternal exposure to sub-lethal heat stresses of 25 °C for 1 h or 28 °C for 48 h negatively affects the developmental duration and percentage of individuals completing overall development in the offspring. However, the egg-hatching rate of the offspring improved with an increase in the maternal treatment temperature^22^. In *Nilaparvata lugens*, exposure to heat stress during either the first instar or adult stage significantly prolongs the developmental period of the eggs of the offspring^23^. For *Drosophila melanogaster*, higher temperatures experienced by the maternal generation result in higher offspring fitness, which may be associated with the accelerated development of the offspring^24^.

In this study, *Sitobion avenae* was selected as the experimental organism. This species, with a short life cycle (average lifespan of 20–30 days under optimal conditions), high reproductive capacity (producing 3–5 nymphs per day) and high outbreak frequency, is a dominant wheat aphid and a major global cereal pest^25,26^. This insect is small in size, with a body length ranging from 1.5 to 3.0 mm, has a large specific surface area, and exhibits rapid heat conduction and heat exchange with the external environment, making it highly sensitive to external temperature changes^27,28^. These characteristics mean that aphid populations can fluctuate significantly in response to climate change, which in turn can have substantial impacts on crop production. Furthermore, the aphid possesses unique telescoping generations^29,30^implying the embryo in this species also contains a developing embryo, which has significant implications for the offspring. Hence, the maternal living environment significantly influences offspring performance.

We examined how thermal stress characteristics influence life history traits of Sitobion avenae across generations, addressing: (1) Will the interaction between different types and frequencies of thermal stress affect the life history traits of this species, and will this effect be similar in the F0 and F1 generations? (2) Will this interaction vary based on the type of thermal stress? (3) Does subjecting the insect to a higher frequency of heat have a greater effect? Based on prior studies, we hypothesize that: (1) Heatwave type and frequency will differentially impact F0 and F1 generations, with distinct effects between the two generations^31^; (2) The interaction effect will vary depending on the type of thermal stress, with prolonged mild stress (34 °C/180 min) causing greater fitness trade-offs than short, intense stress (38 °C/10 min)^32^; (3) Higher frequency (5-day) stress will amplify intergenerational costs^33^.

## Materials and methods

### Study insects

A monoclonal aphid line was implemented in the laboratory to analyze intergenerational effects^34^. This clonal line was acquired from a winter wheat field near Linfen, China (35°55’N, 111°16’E) and then reared as described by Cao et al. (2018)^35^.

### Thermal regimes

The temperature data of the primary occurrence season (May) of wheat aphids in Linfen indicated that the maximum field temperature was approximately 38 °C (Fig. S1A). When field temperature is > 30℃, *S. avenae* experiences thermal stress^36^ and exhibits autonomous heat avoidance behavior^19,20^. During the experimental design phase, we selected six temperature treatments (30, 32, 34, 36, 38, and 40 °C) for preliminary experiments to assess the aphids’ autonomous heat avoidance behavior. Thirty 9-day-old apterous aphids were placed individually in the holes (diameter 5 mm, depth 5 mm) of a honeycomb plate (80 × 80 mm) attached to nylon gauze (200 mesh size) on one side and a transparent plastic board on the other side. The honeycomb plate was placed vertically within a double-glazed container (diameter100 mm, height 100 mm) heated by a glycol bath (Ministat 230-cc-NR; Huber Ltd., Germany; accuracy ± 0.01℃). Aphids were observed from immobility or slow movement to exhibiting autonomous heat avoidance behavior (characterized by pronounced body movements and struggles) using a SONy^®^ video camera (HDR-PJ50E; Sony Corporation, Shanghai.China). For each temperature treatment, 30 aphids were randomly selected. Preliminary results showed no significant avoidance behavior at 30–32 °C within 6 h, while 40 °C caused rapid inactivity, making this temperature unsuitable for evaluating thermal stress effects. For the effective temperatures (34, 36, 38 °C), the average response times were 180.6 ± 10.6 min, 30.6 ± 1.3 min, and 10.1 ± 1.2 min (mean ± SD), respectively. These data directly informed the establishment of heatwave types. Additionally, heatwave frequency primarily consists of consecutive hot days of 1 − 5 days (Fig. S1B). Therefore, we implemented a series of heat events by simulating daily cycles of thermo-periods, with thermal frequency ranging from 1 to 5 days. Finally, nine regimes of three heatwave types (34 °C/180 min, 36 °C/30 min, and 38 °C/10 min) and three frequencies (1, 3, 5 days) were established.

### Test operation

To study the delayed effect of thermal type and frequency on the F0 and F1 generations of *S.avenae*, we designed a full-factorial experiment consisting of the ten treatments: nine thermal regimes and one control (22℃) (Fig. S2). Forty 9-day-old apterous were randomly selected for each treatment in the F0 generation. Additionally, some spare aphids were prepared for each treatment, specifically for the collection of offspring. At the end of the F0 generation, 30 new nymphs were randomly chosen from each treatment in the Fl generation.

The test aphids were placed individually in a 5-mL plastic rearing tube (diameter: 15 mm and length: 70 mm) sealed with a sponge plug at both ends, with a 10-mm slit in the sponge plug at one end used to secure a wheat seedling (50 mm length) as a food source^35^. To ensure that the temperature in the rearing tube matched the set temperature during heat stress, the tubes were preheated in an artificial climate box (RXZ.380B, Jiangnan Co., Ltd.) at 34, 36, and 38 °C for10 min. The test commenced at 08:00, with the test aphids quickly introduced into the preheated feeding tubes. After treatment at the respective temperature for the specified time, the aphids were removed and placed in an artificial climate box (22 °C) to continue feeding until 08:00 the next day. This procedure was repeated for 5 days, resulting in nine thermal treatments. During the treatment period, the relative humidity was maintained at 50 − 60%. The photoperiod was set from 06:00 to 22:00. To ensure the wheat seedlings remained fresh, we replaced them every 2 days.

Delayed effects on the F0 generation: After the experiment commenced, the longevity and fecundity of the test aphids in each tube were checked once daily at 08:00. Newborn nymphs and dead aphids were removed without disturbing the experimental aphids. The longevity was recorded from the start of the experiment until each treated aphid died. The fecundity was the total number of offspring produced from the start of the experiment until their death.

Delayed effects on the Fl generation: Each new nymph was placed in a separate rearing tube and fed continuously on fresh wheat seedlings in an artificial climate box maintained at 22 ºC. The nymphal survival rate, developmental time, longevity, and fecundity of aphids were investigated. Nymphal survival rate refers to the percentage of nymphs still alive by the adult stage. Developmental time was estimated as the duration of the nymphal stage, from birth to adulthood. Longevity was calculated as the time from adult emergence until death. Fecundity was defined as the overall number of nymphs produced by each adult.

### Data analysis

All statistical tests were performed using SPSS version 20 (SPSS Inc.; Chicago, IL, USA) or R 3.5.1 (RStudioInc., 2018). The Shapiro-Wilk test was used to test the normality of the data. The longevity and fecundity of the maternal generation and the developmental time of the F1 generation did not meet the assumptions for normality and was square-root transformed to improve its normality. Therefore, the data for the fitness traits, except nymphal survival rate, were analyzed using parametric tests and a general linear model. Tukey’s method was used for multiple comparisons among heat types or frequencies, and independent-sample t-tests were employed to compare each treatment against the control. The nymphal survival rate was estimated using a Chi-square analysis of a 3 × 3 contingency table, and differences between heat types or frequencies were determined via a separate contingency table with Bonferroni adjustment^35^. Subsequently, 2 × 2 contingency tables were employed to compare each treatment against the control group, with significance levels similarly adjusted using the Bonferroni method. Additionally, demographic parameters, such as the net reproductive rate (*Ro*), generation time (*G*), and intrinsic rate of increase (*r*_*m*_) for each treatment, were calculated as described by Cao et al. (2021)^31^with their means and corresponding 95% confidence intervals estimated via the bootstrap procedure. Multiple comparisons between heat types or frequencies were conducted using the Kruskal-Wallis method, while Mann-Whitney U tests were were used to compare each treatment with the control group.

## Result

### Effects of thermal experience in the F0 generation

#### Longevity

All treatments significantly reduced the adult longevity of the maternal generation (9.6 d in control; *P* < 0.05). The three heatwave types (34 ℃/180 min, 36 ℃/30 min, and 38 ℃/10 min) resulted in significant differences in the contemporary longevity at a 1-day frequency (*F*_2,117_ = 3.584, *P* = 0.031). The most adverse effects were observed under 34 °C/180 min, where the longevity was 3.5 days. However, the difference diminished at frequencies of 3 days (*F*_2,117_ = 1.376, *P* = 0.257) and 5 days (*F*_2,117_ = 1.533, *P* = 0.220).

**Fig. 1:**
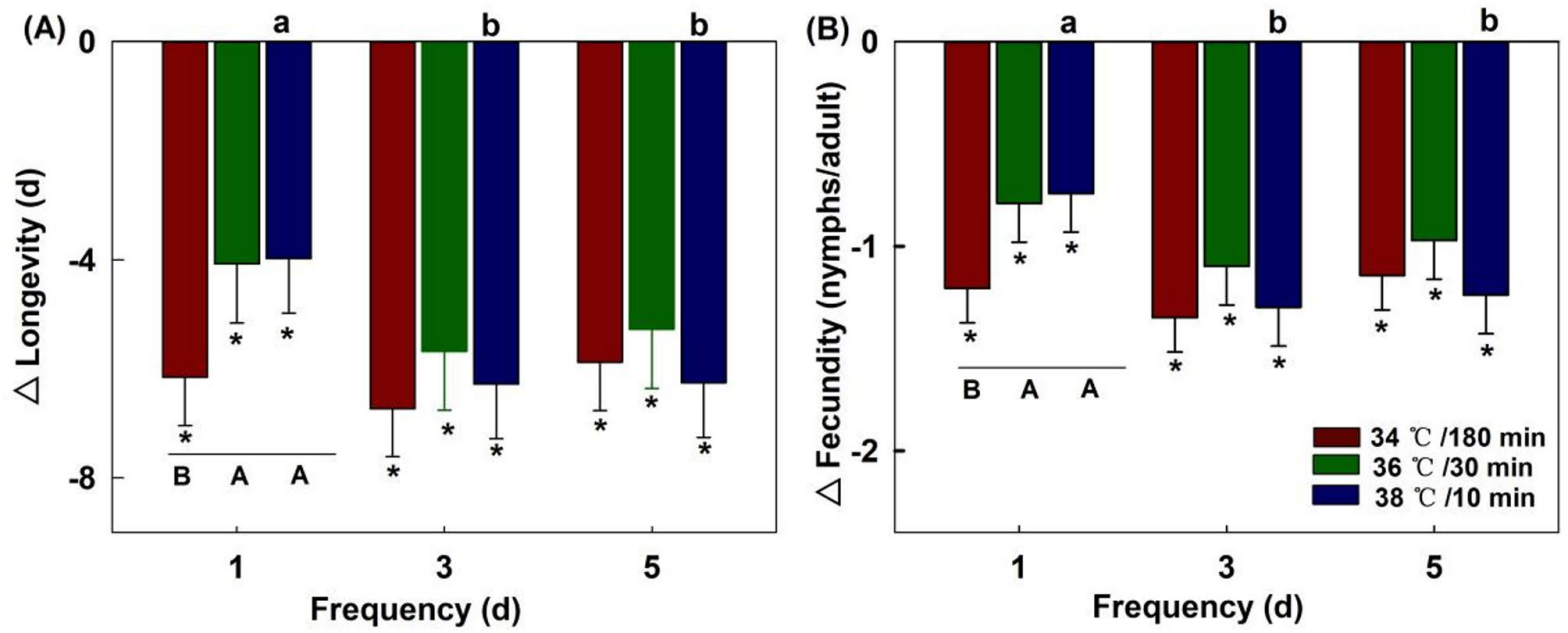
Effects of thermal regimes on the longevity and fecundity in the maternal generation (F0). Each bar represents the mean value and standard error corresponding to the different treatment minus the control group. “*” represent significant difference (*P* = 0.05) between a treatment and control group. Capital letters indicate significant level (*P* = 0.05) among three heatwave types at the same frequency. Lowercase letters indicate significant level (*P* = 0.05) among three frequencies for the same heatwave type.

The frequency of heat stress had no significant effect on the longevity of the maternal generation, which was treated at 34 ℃/180 min (*F*_2,117_ = 1.022, *P* = 0.363) and 36 ℃/30 min (*F*_2,117_ = 1.489, *P* = 0.230). However, the frequency had a significant effect at 38 ℃/10 min (*F*_2,117_ = 5.849, *P* = 0.004). The longevity was shortened from 5.7 days at a frequency of 1 day to 3.4 days frequencies of 3 and 5 days (Fig. 1A).

#### Fecundity

Similar to longevity, all treatments significantly reduced the adult longevity of the maternal generation (11.0 nymphs per adult in control; *P* < 0.05). The three heatwave types showed significant differences in the contemporary fecundity at a frequency of 1 day (*F*_2,117_ = 3.674, *P* = 0.028). The most adverse effects were observed under 34 °C/180 min, where the fecundity was only 2.3 nymphs per adult. However, the difference was not significant at frequencies of 3 days (*F*_2,117_ = 1.090, *P* = 0.340) and 5 days (*F*_2,117_=2.885, *P* = 0.060).

The frequency of heat stress had no significant effect on the fecundity of the maternal generation when treated at 34 ℃/180 min (*F*_2,117_ = 0.957, *P* = 0.387) and 36 ℃/30 min (*F*_2,117_ = 0.868, *P* = 0.422). However, at 38 ℃/10 min, the frequency had a significant effect (*F*_2,117_ = 7.407, *P* = 0.001), with fecundity decreasing from 6.0 to 2.1 nymphs per adult as the frequency increased (Fig. 1B).

### Effects of thermal experience in the F1 generation

#### Nymphal survival

Compared with the nymph survival rate of 80% in the offspring of the control group, all stress treatments on the maternal generation reduced the nymph survival rate of the offspring. The three heatwave types showed no significant differences in nymphal survival rates in the F1 generation after the maternal were exposed to these stresses for only 1 day (*χ*^*2*^ = 3.732, *df* = 2, *P* = 0.155). However, significant effects were observed at frequencies of 3 days (*χ*^*2*^ = 15.210, *df* = 2, *P* < 0.001) and 5 days (*χ*^*2*^ = 10.167, *df* = 2, *P* = 0.006). Regardless of the frequency of maternal exposure to thermal stress, the treatment at 34 °C/180 min had minimal adverse effect on the nymphal survival rate in the F1 generation.

**Fig. 2:**
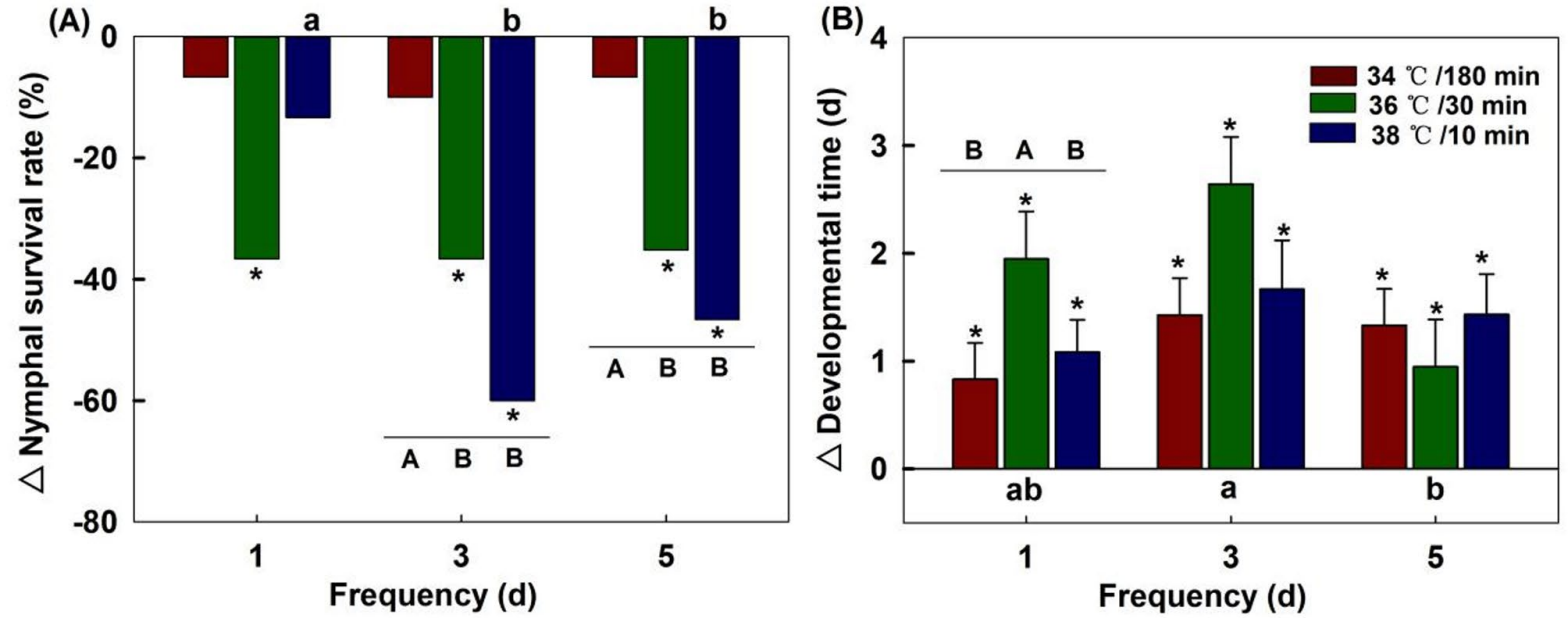
Effects of maternal thermal regimes on the survival and development in the offspring generation (F1). Each bar represents the mean value or standard error corresponding to the different treatment minus the control group. “*” represent significant difference (*P* = 0.05) between a treatment and control group. Capital letters indicate significant level (*P* = 0.05) among three heatwave types at the same frequency. Lowercase letters indicate significant level (*P* = 0.05) among three frequencies for the same heatwave type.

The frequency of maternal exposure had no significant effect on the nymphal survival rate in the F1 generation under 34 ℃/180 min (*χ*^*2*^ = 0.111, *df* = 2, *P =* 0.946) and 36 ℃/30 min (*χ*^*2*^ = 0.295, *df* = 2, *P* = 0.863). However, significant effects were observed under 38 ℃/10 min (*χ*^*2*^ = 14.444, *df* = 2, *P* = 0.001), with the survival rates sharply decreasing from 67% at a frequency of 1 day to 20% at a frequency of 3 days (Fig. 2A).

#### Development

Compared to the 8.7 d developmental time in control offspring, all maternal stress treatments significantly prolonged the developmental time of offspring (*P* < 0.05). The three heatwave types affected the developmental time of the F1 generation when the maternal generation was exposed to these stresses for only 1 day (*F*_2,52_ = 4.588, *P* = 0.015). Specifically, the developmental time was significantly prolonged to 9.6 days at 36 ℃/30 min compared with that under the other two heat stresses conditions. Moreover, this prolonged effect persisted but was not significant at a frequency of 3 days (*F*_2,37_ = 2.337, *P* = 0.111) and completely disappeared at a frequency of 5 days (*F*_2,42_ = 0.589, *P* = 0.560).

The frequency of maternal exposure did not have a significant effect on developmental time in the F1 generation under 34 ℃/180 min (*F*_2,62_ = 1.475, *P* = 0.237) and 38 ℃/10 min (*F*_2,33_ = 0.806, *P* = 0.455). However, a significant effect was observed at 36 ℃/30 min (*F*_2,36_ = 4.453, *P* = 0.019), with the longest development time of offspring recorded at 10.3 days when the maternal heat exposure frequency was 3 days (Fig. 2B).

#### Longevity

**Fig. 3:**
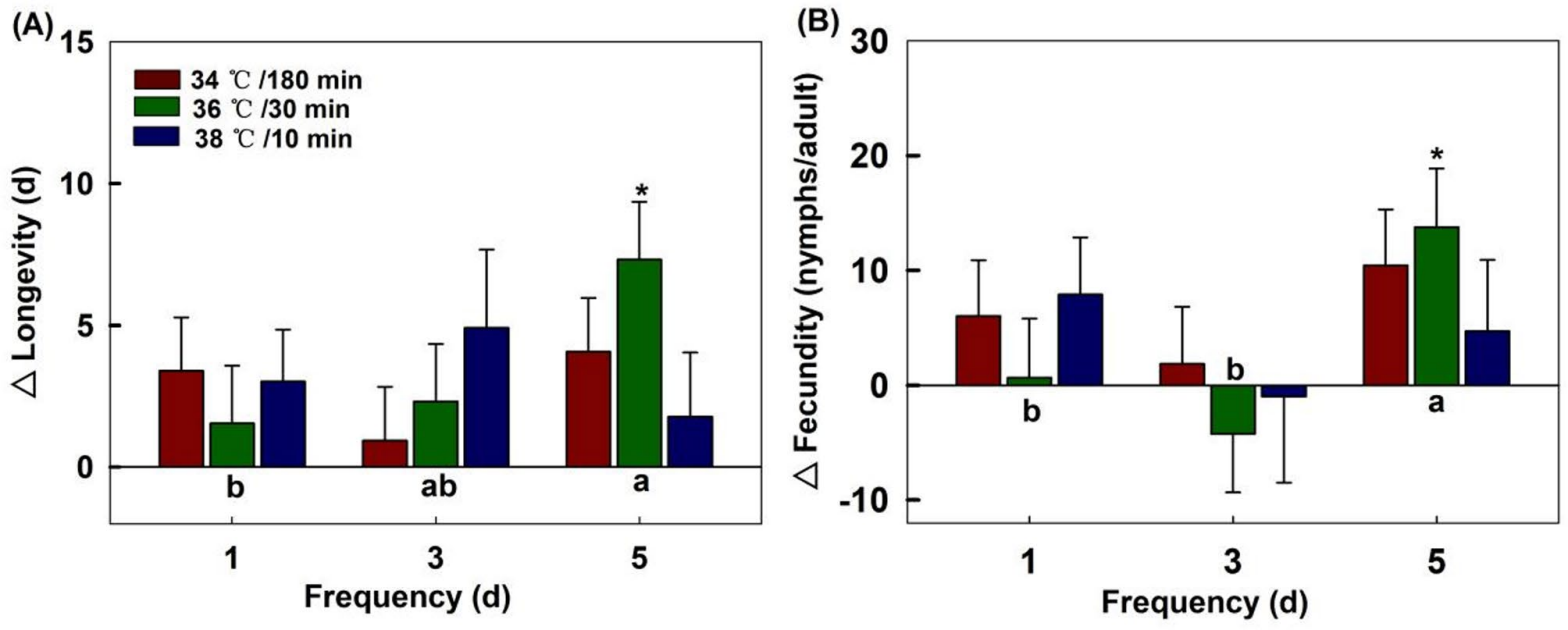
Effects of maternal thermal regimes on the longevity and fecundity in the offspring generation (F1). Each bar represents the mean value and standard error corresponding to the different treatment minus the control group. “*” represent significant difference (*P* = 0.05) between a treatment and control group. Capital letters indicate significant level (*P* = 0.05) among three heatwave types at the same frequency. Lowercase letters indicate significant level (*P* = 0.05) among three frequencies for the same heatwave type.

Compared to the offspring longevity of 9.9 d from the control group, all maternal stress treatments exerted stimulatory effects on longevity. The three heatwave types had no significant effect on longevity in the F1 generation when the maternal generation was exposure for 1 day (*F*_2,52_ = 0.278, *P* = 0.758), 3 days (*F*_2,42_ = 0.883, *P* = 0.422), and 5 days (*F*_2,42_ = 2.833, *P* = 0.070). The frequency of maternal exposure did not have a significant effect on the longevity of the F1 generation under 34 ℃/180 min (*F*_2,62_ = 1.285, *P* = 0.284) and 38℃/10 min (*F*_2,33_ = 0.429, *P* = 0.655). However, an effect was discovered at 36 ℃/30 min (*F*_2,36_ = 3.172, *P* = 0.048), with the longest longevity of offspring recorded at 17 days when the maternal generation was exposed for 5 days (Fig. 3A).

#### Fecundity

Compared to the offspring fecundity of 21.0 nymphs per adult aphid from the control group, all treatments except for the exposure of the maternal generation to 36 °C/30min and 38 °C/10min for 3 days, had a stimulating effect on the offspring’s reproduction. The three heatwave types that the maternal generation was exposed to at frequencies of 1 day (*F*_2,52_ = 0.575, *P* = 0.566), 3 days (*F*_2,37_ = 0.727, *P* = 0.490), and 5 days (*F*_2,42_ = 0.790, *P* = 0.461) had no significant effect on fecundity in the F1 generation.

The frequency of maternal exposure did not have an effect on fecundity in the F1 generation under 34 ℃/180 min (*F*_2,62_ = 1.270, *P* = 0.288) and 38 ℃/10 min (*F*_2,33_ = 0.558, *P* = 0.578). However, a significant effect was found under 36 ℃/30 min (*F*_2,36_ = 4.549, *P* = 0.017), where the highest fecundity of offspring was recorded at 35.0 nymphs per adult when the maternal generation was exposed for 5 days (Fig. 3B).

#### Demographic parameters

**Fig. 4:**
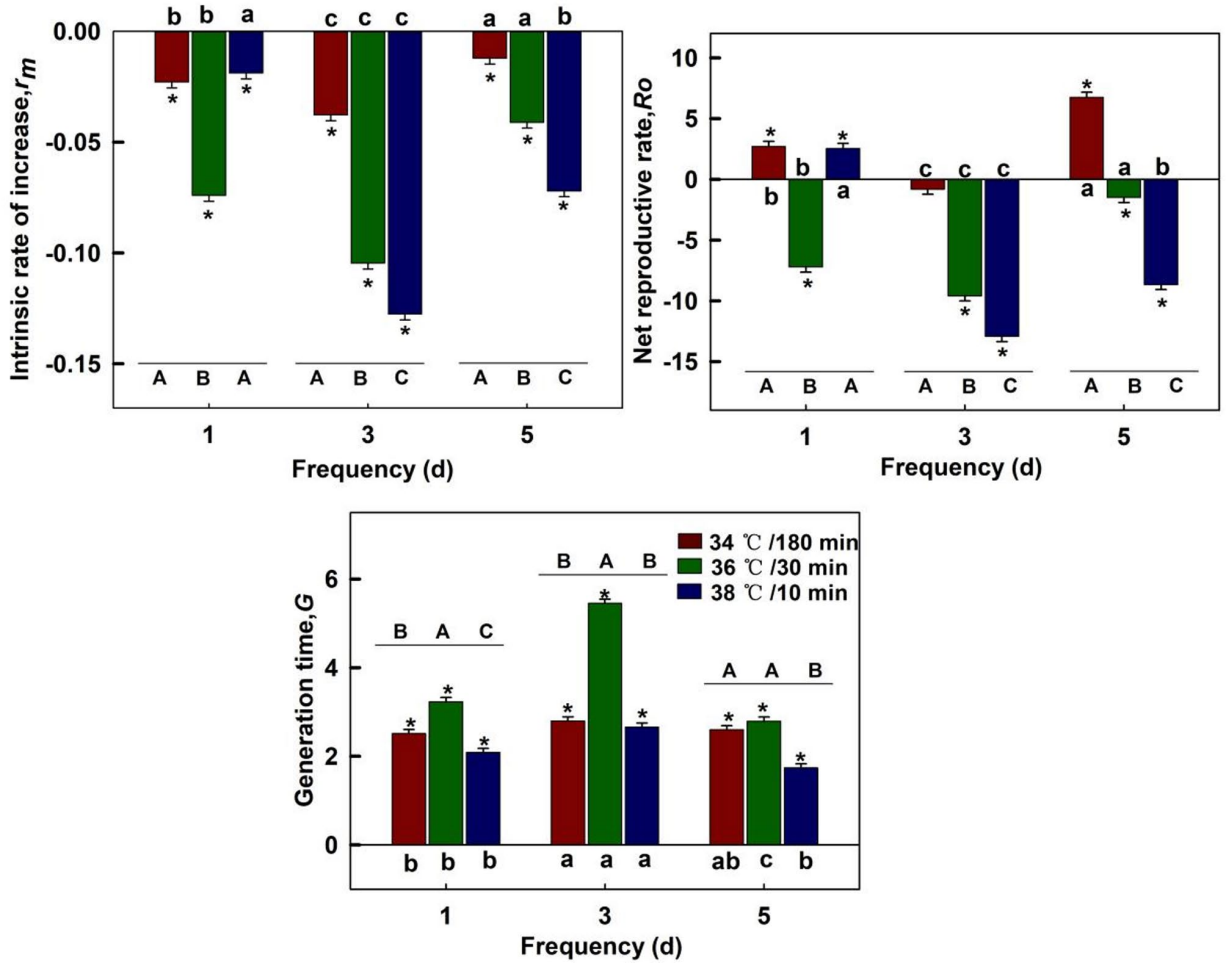
Effects of maternal thermal regimes on demographic parameters in the offspring generation (F1). Each bar represents the mean value and standard error corresponding to the different treatment minus the control group. ^"*"^ represent significant difference (*P* = 0.05) between a treatment and control group. Capital letters indicate significant level (*P* = 0.05) among three heatwave types at the same frequency. Lowercase letters indicate significant level (*P* = 0.05) among three frequencies for the same heatwave type.

Compared with the demographic parameters of the offspring from the control group, maternal stress exposure significantly prolonged the mean generation time (*G*) and reduced the intrinsic rate of increase (*r*_*m*_) in the offspring population (*P* < 0.05). The three heatwave types of maternal exposure at frequency 1, 3, and 5 days had an effect on demographic parameters in the F1 generation. The demographic parameters of the offspring when the maternal was exposed to 34 °C/180 min were more favorable than those under the other two heat stresses, regardless of the frequency of 1, 3, or 5 days. However, the offspring performance was poor under all three stresses after a 3-day maternal exposure frequency (Fig. 4).

## Discussion

Extensive research has explored the effects of thermal stress on life history traits of insects, including their growth, development, survival, and reproduction^37–39^. On hot days, insects experience high temperatures and then engage in avoidance behaviors by moving to cool microhabitats such as vegetation covers and canopy shades, plays helps the insects to counter thermal stress^15,18^. However, previous studies that simulated thermal events primarily focused on external high-temperature changes affecting the life activities of insects^40–42^with limited focus on heat-escape responses by insects^32^. The findings of the present study revealed that aphids exhibit varying levels of sensitivity to different high temperature conditions, and the three heatwave types associated with behavioral responses under various frequency lead to more complex delayed effects.

The three heatwave types significantly affected maternal life history response only at a frequency of 1 day, and the severity of the influence on maternal longevity and fecundity followed the order of 34 °C/180 min > 36 °C/30 min > 38 °C/10 min. This indicates that mild heat, including 34 °C, causes the most damage as it takes longer for the insects to recognize and respond to the discomfort. This phenomenon can be explained by the accelerated metabolic rate under this treatment, resulting in a more rapid consumption of nutrients stored in the body, thereby reducing support for reproduction and lowering the number of offspring^43,44^. In the remaining two treatments, the test insects responded more quickly to escape the heat, thus mitigating the damage. Additionally, as the frequency increased, the differences in maternal fitness traits of longevity and fecundity among the three heatwave types diminished. This finding aligns with that of previous studies indicating that thermal damage is closely related to the frequency of high temperatures^45–47^.

For offspring, maternal thermal experience primarily influenced survival and development. These findings align with those of previous research on maternal effects, in which temperature stress in the F0 generation influenced the survival and development fitness of the Fl generation in aphids^31^. This phenomenon is closely associated with telescopic reproduction^48,49^. These findings align with those of previous reports on other organisms^50,51^indicating a transgenerational effect, where environmental effects experienced by the maternal generation may directly influence the offspring. The demographic parameters of the offspring, when the maternal generation was exposed to 34 °C/180 min, exhibited superiority over those of the other two heat stresses (36 °C/30 min and 38 °C/10 min), regardless of the heatwave frequency. This may be associated with metamorphic modularity^52,53^ or compensatory growth^54,55^which buffer the damage caused by 34 °C, and this may reduce the threat of 34 °C compared with 36 °C and 38 °C. Notably, the F₁ generation exhibited a distinct response to maternal 36 °C/30 min stress compared to 34 °C and 38 °C. While 34 °C (prolonged, low-intensity) and 38 °C (short-term, high-intensity) triggered “gradual acclimation“^33,46^ and “rapid stress response“^8,40^ strategies, respectively—both maintaining relatively stable resource allocation in F₁ and converging in response patterns—36 °C (moderate duration and intensity) fell within an “acclimation-stress blind zone“^31,32^. This led to maternal resource depletion, developmental disruption in F1, and ultimately, unique transgenerational effects. Regarding thermal frequency, maternal exposure for 3 days resulted in the worst offspring population performance compared with that for 1 and 5 days. This indicates that a longer frequency for the mother does not necessarily mean a greater effect on the offspring. This explains that the damage caused by thermal frequency of 1 day was minimal, whereas the damage caused by frequency of 5 days was extensive, resulting in little variation in the fitness of the surviving maternal individuals. However, after thermal frequency of 3 days, some maternal insects with reduced fitness result in offspring with the poorest population performance.

This study reveals asymmetric effects of heat stress types and frequencies on the fitness of maternal and offspring generations of *Sitobion avenae*, providing critical insights for biological risk assessment under climate change. Consistent with our hypothesis, prolonged mild stress (34 °C/180 min) showed the most detrimental effects on maternal fitness, likely due to delayed heat-avoidance behavior and exacerbated metabolic costs^43^. However, the offspring response was inconsistent with the hypothesis: the offspring population parameters were optimal when the maternal generation experienced the 34 °C/180 min treatment owing to metamorphic modularity^52,53^ or compensatory growth^54,55^. Notably, intermediate frequency (3-day, rather than the expected 5-day) caused maximal offspring fitness decline, revealing nonlinear frequency-generation interactions. This finding provides a key insight: a 3-day heat stress frequency represents a critical threshold for offspring fitness and should be incorporated into regional pest early-warning models. For example, in wheat-growing regions such as Linfen, if daily temperatures reach or exceed 34 °C for three consecutive days, subsequent populations of *Sitobion avenae* may experience short-term suppression due to a sharp decline in offspring survival. This offers a scientific basis for field monitoring and pest management decisions, reducing unnecessary pesticide applications. In conclusion, to more accurately predict aphid responses to heat stress, it is essential to consider both the type and frequency of heatwaves, and the delayed effects of heat stress on offspring should also be included in predictive analyses.

## Electronic supplementary material

Below is the link to the electronic supplementary material.

Supplementary Material 1

## Data Availability

The data used to support the findings of this study are available from the corresponding author upon request.
