# Supplementary material for: Heatwave types and frequency alter multigenerational ecological response of wheat aphids
Source: Sci Rep. 2025 Aug 3;15:28307. doi: 10.1038/s41598-025-13097-x (PMC12319083; doi:10.1038/s41598-025-13097-x)
Supplement: Supplementary file 1 — Supplementary Material 1 [file 41598_2025_13097_MOESM1_ESM.doc]

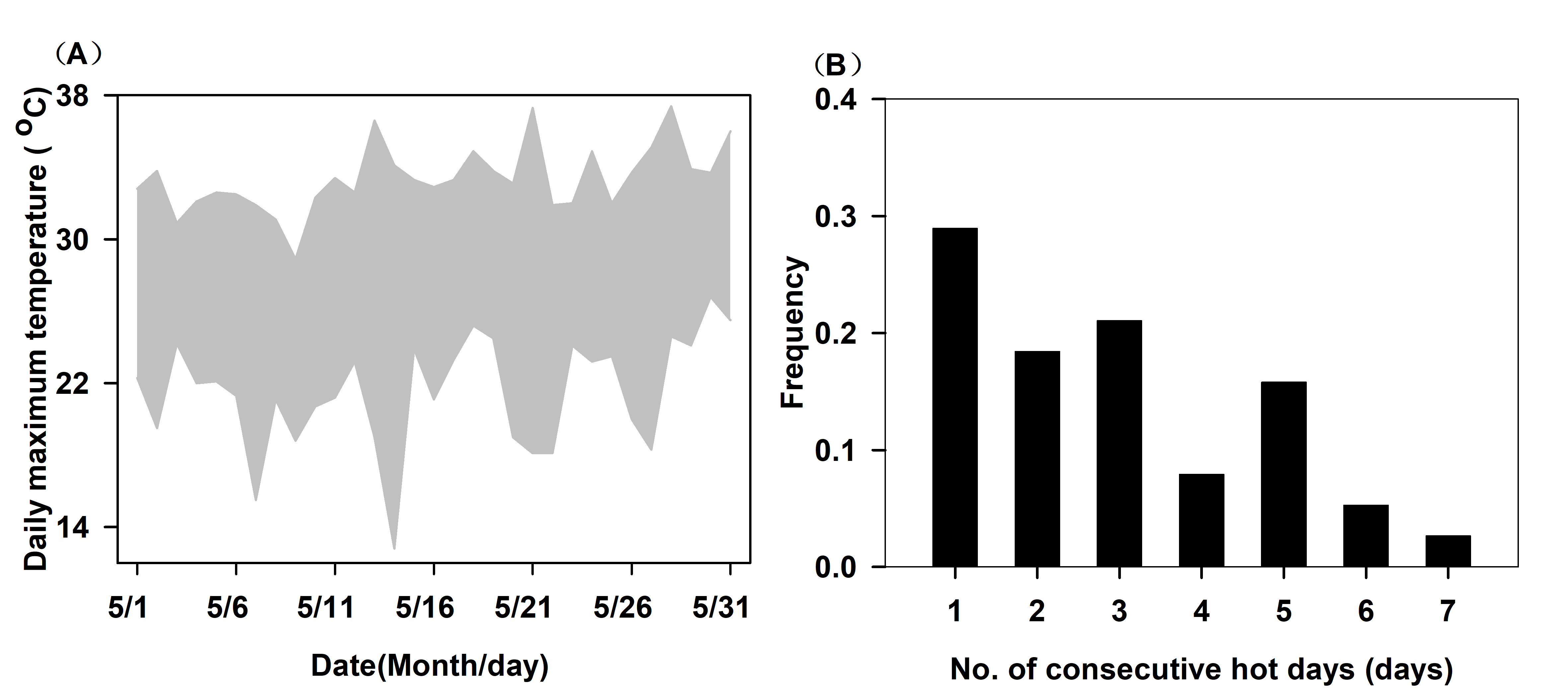
Fig. S1 (A) Variation in daily maximum temperature at Linfen in May from 2010 to 2018. (B) Frequency of consecutive hot days of a hot event (daily maximum temperature ≥ 30°C) from 2010 to 2018. Temperatures records were downloaded from the China Meteorological Data Sharing Service System.


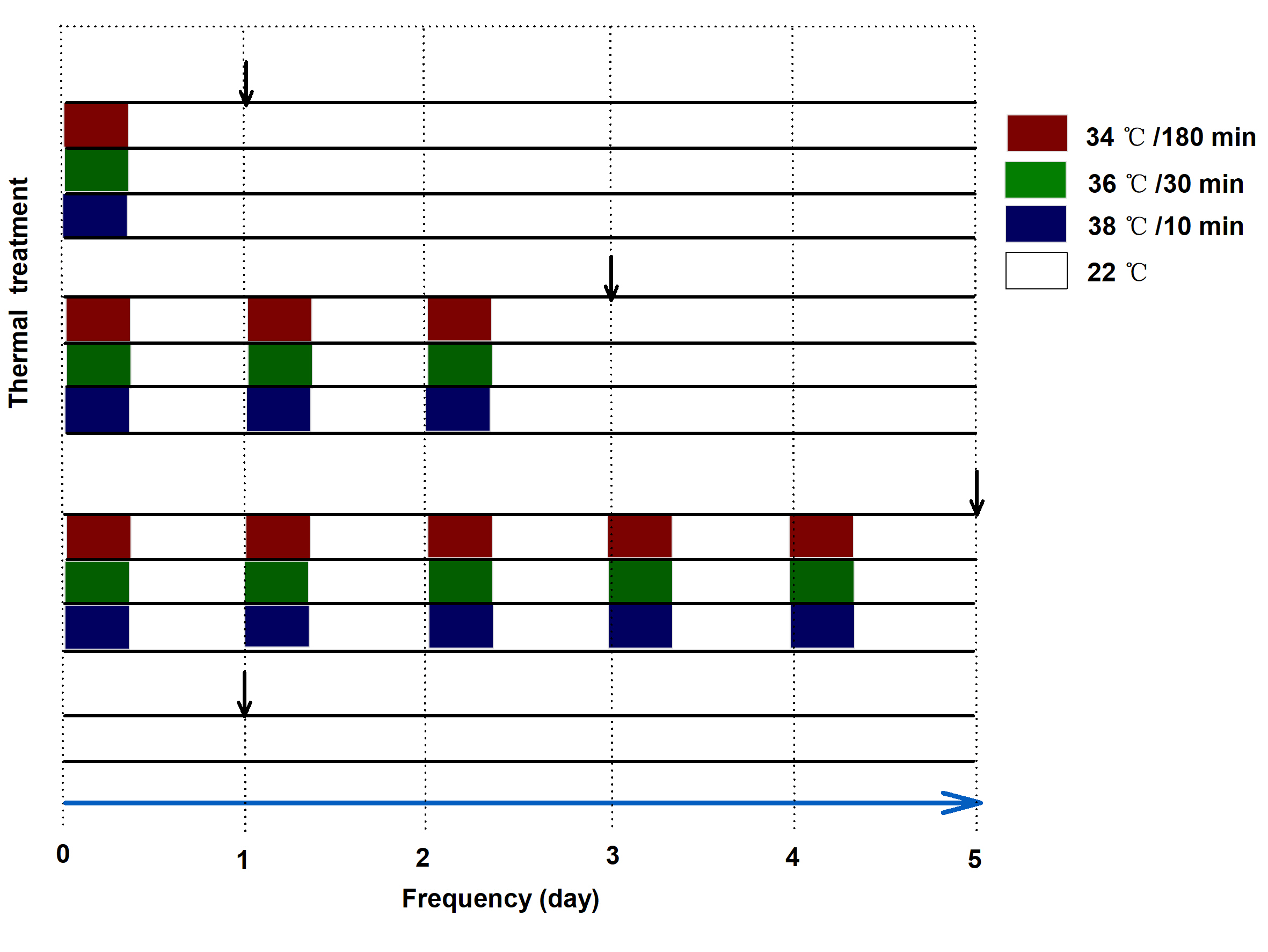


Fig. S2 Experimental design.
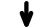
indicates offspring sampling point. “0” indicated when the experiment started.
